# Correction: Hou, J., et al. Anti-Inflammatory Effects of Aurantio-Obstusin From Seed of *Cassia obtusifolia* L. Through Modulation of the NF-κB Pathway. *Molecules* 2018, *23*, 3093

**DOI:** 10.3390/molecules24040745

**Published:** 2019-02-19

**Authors:** Jingyi Hou, Yu Gu, Shuai Zhao, Mengqi Huo, Shifeng Wang, Yanling Zhang, Yanjiang Qiao, Xi Li

**Affiliations:** 1 Research Center of Traditional Chinese Medicine Information Engineering, School of Chinese Materia Medica, Beijing University of Chinese Medicine, Beijing 100102, China; hjy_2016@126.com (J.H); riberyguyu@163.com (Y.G.); 20170931863@bucm.edu.cn (S.Z.); 20150931715@bucm.edu.cn (M.H.); alana6268@126.com (S.W.); xixili1994@163.com (X.L.); 2 Beijing Key Laboratory of Traditional Chinese Medicine Basics and New Drug Research, Research Center of Traditional Chinese Medicine Information Engineering, Beijing University of Chinese Medicine, Beijing 100102, China

The authors wish to make the following correction to their paper [1]. The concentration of Aurantio-obtusin labeled was inaccurate in Figure 5 and Figure 6. The correct versions of Figure 5 and Figure 6 are as follows:

The concentrations of aurantio-obtusin in Figure 5 and Figure 6 were tested at micromole level, instead of millimole in the original version, this change does not affect the scientific results. The manuscript will be updated and the original will remain online on the article webpage. The authors would like to apologize for any inconvenience caused to readers by these changes.

## Figures and Tables

**Figure 5:**
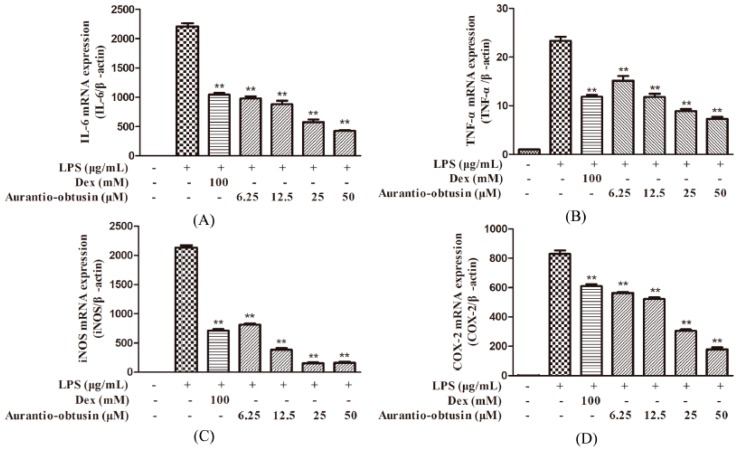
Effect of aurantio-obtusin on LPS-stimulated mRNA expression of IL-6, TNF-α, iNOS, and COX-2. RAW264.7 cells were pre-incubated with various concentrations of aurantio-obtusin (6.25–50 μM) for 2 h, followed by stimulation with LPS (0.2 μg/mL) for 24 h. The mRNA expression of IL-6 (**A**), TNF-α (**B**), iNOS (**C**), and COX-2 (**D**) was analyzed using real-time RT-PCR. The data are presented as mean ± SDs (*n* = 3). **p < 0.01 represents significance when compared to LPS-only treated cells.

**Figure 6:**
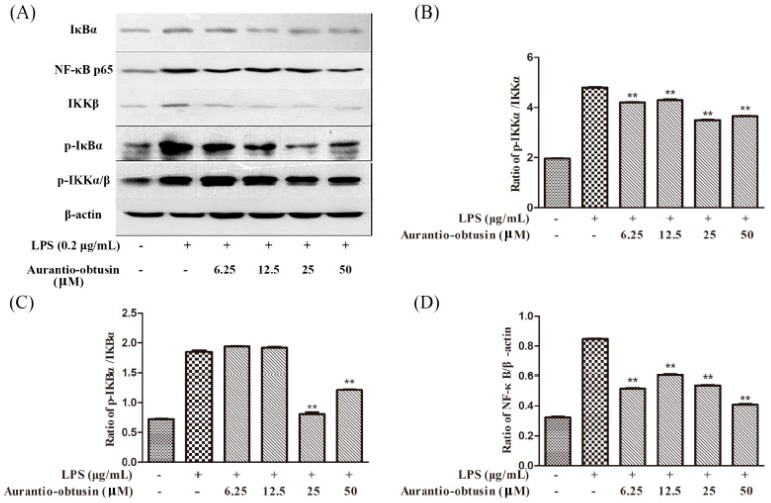
Effects of aurantio-obtusin on the expression of proteins associated with inhibition of NF-κB in LPS-stimulated RAW264.7 mouse macrophages. RAW264.7 cells were pre-incubated with various concentrations (6.25–50 μM) of aurantio-obstusin for 2 h, and stimulated with LPS (0.2 μg/mL) for 12 h. The β-actin protein was used as the internal control. Expression of proteins associated with the inhibition of NF-κB was detected by a western blot analysis (**A**). Comparison of the levels of phosphorylated protein relative to the levels of their non-phosphorylated counterparts in the grey scale: p-IKKα/ IKKα (**B**) and p-IKBα/ IKBα (**C**). Comparison of the levels of p65 relative to the levels of their actin counterparts in the grey scale (**D**). **p < 0.01 represents significance when compared to LPS-only treated cells.
